# The Cost of Medical Education

**Published:** 1908-09-05

**Authors:** 


### THE COST OF MEDICAL EDUCATION.

To those who have already decided to send their sons into the medical profession, no less than to those who are still weighing the matter in their minds, a few remarks on the cost of medical education and the probable duration of the course of study are likely to prove of interest. The question of ways and means during the long years of studentship lies close at hand, and at the present time of financial depression it is liable to assume even greater significance than the question of future status and remuneration. The initial cost of putting a young man in the way of earning his living as a doctor is only too often a heavy strain upon the family exchequer; and it is better to look things squarely in the face at the very beginning, rather than to discover, when it is too late to turn back, that the first rough estimates of time and expenditure were based upon the optimistic programme of a medical school prospectus, and not upon the average performance of the average medical student.

Firstly, with regard to the time occupied. Five years is the minimum period—counting from the time of registration as a medical student—which is laid down by the General Medical Council; and there are men even to-day, who, by system and diligence and examinational skill, succeed in qualifying at the end of that time. But their number is few, and it is indeed doubtful whether such rapidity is always desirable. The sharp, industrious youth, who at twenty-one is a qualified practitioner, may regret afterwards the unbroken sequence of examination triumphs which punctuated his single-minded devotion to the subjects of the curriculum. After all, examinations are uncertain things and merely approximate tests of knowledge and competence. To pass each one at the earliest opportunity is an excellent ideal, and the school prospectuses are no doubt wise in assuming that it will be attained; but parents would do well to expect something less than this, and to be satisfied if their sons obtain the London Conjoint Board Diploma within six years from the commencement of medical study, or the Cambridge or the London M.B. degree before the end of six years and a half.

We have considered the time: we now come to the question of money. It is often said that it costs a thousand pounds to become a doctor, and this is perhaps nearer the truth than most current generalisations. The expenses of medical education, of course, vary within wide limits. The upper limit is naturally impossible to define, and it would be absurd to consider it. What we have to consider is, firstly, the lowest figure on which it is possible with extreme economy and rigid self-denial for a poor man to become qualified; and, secondly, the approximate sum which a parent of good social position and moderate means should be prepared to put aside for the maintenance and medical education of a son who has just left a good public school, and is already endowed with a reasonable share of diligence, common sense, and ability.

In the case of the poor student, to whom every penny and every minute is a matter for anxious thought, it is well to point out at once that a first-class medical education leading to the ultimate possession of a good medical degree is more easily to be obtained for a small outlay in the university cities of Scotland than anywhere else in the Kingdom. Living is uniformly cheaper there, fees are for the most part lower, and opportunities for economising in a variety of ways are more accessible and more popular; while the munificence of Mr. Andrew Carnegie has been directed towards the assistance of this very type of student.

In London, on the other hand, where medical education and maintenance is probably more costly than in any other city within the three kingdoms except Oxford and Cambridge, it is hardly possible (in the absence of scholarships) for a student, even with the most rigorous economy, to keep himself in food, lodging, clothing, books, and other necessary equipment, and to pay his hospital and examination fees, on an income of less than £100 a year. Thus, if we include the cost of diplomas and of registration, but make allowance neither for failures at examinations nor for anything but the scantiest of amusements and holidays, our poor student must reckon on a total expenditure of at least £550 during his five years in London, or of £600 if he undertakes the 5½ years' course which is now the minimum required by the University of London for its M.B. degree. To supplement his income by acting as a part-time dispenser, by undertaking stray translations and other scraps of literary hack-work, or even by preparing microscopical specimens for an exiguous recompense, is, in our opinion, to distract attention from the work in hand, and can only be justified by the harshest necessity.

As for the more ordinarily situated medical student—he whose father can afford to keep him in tolerable comfort during the six years or so of studentship—the sum which should be set aside for his living and educational expenses cannot well be less than £200 a year. The composition fees for the full course at London hospitals vary from about £120 to £170; but there will be other necessary payments as time goes on, for such purposes as anatomical "parts," operative surgery courses, laboratory apparatus and club subscriptions; while a set of bones, a good microscope, and a gradually increasing library of text-books are all inevitable sources of expenditure; and there are some students to whom the services of a good "coach" would seem at one time or another to be well-nigh indispensable. Living at home, when the parents dwell in London or its suburbs, is a means of reducing expense, and appears to relieve some parents of a certain form of anxiety; but there are students who are genuinely unable to study so profitably at home as in the privacy of lodgings, shared, it may be, with a fellow-worker of regular habits. Three of the London hospitals, namely, St. Bartholomew's, Guys, and Middlesex, have residential colleges, and parents would be well advised to consider the advantages of these, during part at least of the curriculum. The cost of living is not very different from that in fair lodgings in one of the "students' quarters." Living in a doctor's family depends on the doctor and his family, and may or may not be a success.

Parents who can afford it should, we consider, send their sons for the first three years of their medical course to one of the older universities; this usually means an outlay of more than £200 a year for the time being, and some extension of the total time of study. Lastly, we may refer to a matter of expenditure which is likely to arise at the end of the curriculum. The parent may then learn that the newly qualified son, whom he has supported for twenty-four years, so far from starting off at last to practise medicine for gain, now proposes to hold one or more resident hospital appointments and to remain a burden, albeit somewhat lightened, to the family exchequer. The course of medical education is indeed long, and the cost is heavy; and it is only right that those whose duty or whose pleasure it will be to pay the piper should have some notion beforehand of the kind of tune that is going to be played and of the probable length of the bill.

